# Socioeconomic status is associated with reduced lung function in China: an analysis from a large cross-sectional study in Shanghai

**DOI:** 10.1186/s12889-016-2752-3

**Published:** 2016-02-01

**Authors:** Adam W. Gaffney, Jing-qing Hang, Mi-Sun Lee, Li Su, Feng-ying Zhang, David C. Christiani

**Affiliations:** 1 Massachusetts General Hospital, 55 Fruit Street, Boston, MA 02114 USA; 2 Shanghai Putuo District People’s Hospital, Shanghai, China; 3 Department of Environmental Health, Harvard T.H. Chan School of Public Health, 677 Huntington Ave, Boston, MA 02115 USA; 4 Harvard Medical School, Boston, MA USA

**Keywords:** Socioeconomic factors, Respiratory disease, Environmental health, Epidemiology, China, Pulmonary function

## Abstract

**Background:**

An inverse association between socioeconomic status and pulmonary function has emerged in many studies. However, the mediating factors in this relationship are poorly understood, and might be expected to differ between countries. We sought to investigate the relationship between socioeconomic status and lung function in China, a rapidly industrializing nation with unique environmental challenges, and to identify potentially-modifiable environmental mediators.

**Methods:**

We used data from the Shanghai Putuo Study, a cross-sectional study performed in Shanghai, China. Participants completed a questionnaire and spirometry. The primary exposure was socioeconomic status, determined by education level. The primary outcomes were FEV_1_ and FVC percent predicted. Multiple linear regressions were used to test this association, and the percent explained by behavioral, environmental, occupational, and dietary variables was determined by adding these variables to a base model.

**Results:**

The study population consisted of a total of 22,878 study subjects that were 53.3 % female and had a mean age of 48. In the final multivariate analysis, the effect estimates for FEV_1_ and FVC percent predicted for low socioeconomic status (compared to high) were statistically significant at a p-value of <0.01. Smoking, biomass exposure, mode of transportation to work, a diet low in fruits or vegetables, and occupational category partially attenuated the relationship between SES and lung function. In a fully-adjusted age-stratified analysis, the socioeconomic disparity in lung function widened with increasing age.

**Conclusions:**

We found cross-sectional evidence of socioeconomic disparities in pulmonary function in Shanghai. These differences increased with age and were partially explained by potentially modifiable exposures.

## Background

An inverse association between socioeconomic status (SES) and lung function has been described in the epidemiological literature for decades [1]. Whether SES is measured by individual education [2–7], parental status [8], income [3], occupation [2, 9], or residential area deprivation [2], a social gradient in pulmonary function emerges in most studies.

However, the pathways through which low SES cause reduced lung function remain obscure. In one review, Hegewald and Crapo propose a number of putative factors linking SES and pulmonary function, including prenatal exposures, air pollution, nutritional factors, and occupational exposures [1]. Most likely, a multitude of environmental intermediaries plays a role in the complex relationship between SES and lung function [9]. Additionally, it is plausible that environmental factors are location-specific and differ among high, middle, and low-income countries, which contend with diverse environmental health challenges.

The majority of studies to date on this topic, however, have been performed in high-income countries [1]. While some studies have been performed in low and middle-income countries [10, 11], fewer have been performed in China, a rapidly industrializing nation [12] with unique environmental hazards and a large burden of respiratory disease [13]. For instance, though some studies have identified an association between education and airflow obstruction in China [14–16], we are not aware of any studies that have sought to identify environmental mediators between SES and lung function in China. Given the association between reductions in pulmonary function and mortality [17, 18], identification of these potentially modifiable mediators could have significant public health implications.

We therefore undertook this study using data from the Shanghai Putuo Cohort Study, a large scale population-based cross-sectional study in Shanghai, China to investigate the impact of SES on lung function in Shanghai, and to consider a number of environmental, occupational, and dietary exposures as potential mediators in this relationship.

## Methods

### Study design and data collection

The Shanghai Putuo study is a population-based cross-sectional study performed in Shanghai, China. It is a collaboration between the Harvard School of Public Health and the Shanghai District Peoples Hospital. The Institutional Review Boards of both institutions approved the study (Harvard T.H. Chan School of Public Health IRB Protocol #CR-14777-01). The design of the study has been previously published in detail [19–21], but is briefly summarized here. Study subjects were recruited on the basis of random selection using census tract data between August 2007 and January 2010. There were no exclusion criteria. Subjects were questioned in person by interviewers who were trained and tested in the administration of the study questionnaire. These questionnaires included a wide range of social, demographic, occupational, environmental, and dietary queries.

For SES, our primary exposure of interest, we used education instead of current income in order to reduce the problem of reverse causality. Education was self-reported by study participants (“illiterate” (1), “primary” (2), “junior middle school” (3), “senior middle school” (4), “senior training school and high school” (5), and “college or above” (6)). This was simplified into a three category variable that approximates educational strata in the United States: (A) low: levels 1 to 3 (extending up to children age 13), (B) intermediate: levels 4–5 (typically ages 13–18), and (C) high: level 6 (ages 18+).

**Fig. 1:**
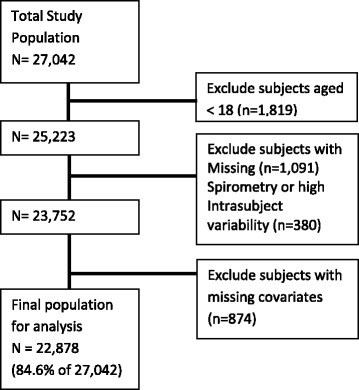
Flowchart of study population formation

A total of 37,690 subjects were selected at random for recruitment. Of these, 27,042 agreed to participate in the study and provided written consent (see Fig. 1). Information on gender and age (<18 or ≥ 18) were available on some (7291 of 10,648) of those who declined to participate. Of these, 7016 individuals were ≥ 18 years, and of these 3713 (52.9 %) were male and 3303 (47.1 %) were female. Of the 27,042 participants, 1819 were less than 18 years of age and were excluded. Of the remaining subjects, 1091 had missing spirometry and were excluded. An additional 380 subjects with high within-subject variability as assessed by a coefficient of variation for FVC of greater than 20 % were also excluded. Of the remaining subjects, 874 had missing covariate data, and were excluded. This left a total of 22,878 subjects, or 84.6 % of the parent study population of 27,042, for our final analysis.

### Spirometry

Measurement of FEV_1_ and FVC was performed according to American Thoracic Society guidelines [22] by a trained technician using a hand-held spirometer (Micro plus, Micro Ltd., Rochester UK) as previously described [19]. Subjects performed spirometry while in a sitting position. All study subjects performed at least three efforts, and the highest FEV_1_ and FVC was used from these measurements. To be considered acceptable, the FVC had to be sustained for no less than six seconds. In order to assess for within-subject variability, we calculated a coefficient of variation (CV) using the standard deviation of the FVC divided by its mean × 100. A CV of 20 % or less was considered acceptable, a threshold that has been used in other studies [19, 23, 24]. As described above, subjects that did not meet this threshold were subsequently excluded from our analysis. We used a validated prediction equation for the adult Chinese population to calculate the FEV_1_ and FVC percent predicted for each study subject [25].

### Statistical analysis

The primary outcome measures were FEV_1_ percent predicted and FVC percent predicted. The primary exposure was SES, which was treated as a categorical variable as described above. We additionally selected covariates for inclusion in our model which we posited might function as putative intermediaries between SES and lung function. Smoking was treated both as a categorical variable (current, former, or never) and in pack-years of smoking history. Second-hand smoke exposure (SHS) and home biomass exposure were treated as binary variables (ever versus never exposed). Subjects were considered exposed to biomass if they reported any previous exposure to solid fuels (which included coal and/or biomass) for cooking and/or heating in the home. Mode of transportation to work, which has been associated with lung function in this study population in work by our group [21], was treated as a categorical variable (bus without air conditioning, bus with air conditioning, bicycle, scooter, taxi, company car, private care, train, subway, walking, none, or multiple forms). A diet low in fruit or vegetable consumption (<1 serving/daily in either (a) fruits or in (b) both cooked and raw vegetables) was used as a surrogate for low-antioxidant consumption, which has been associated with reduced lung function [26]. Occupational status was treated as a categorical variable (farmer, worker, professional, administrator, services, household, retired, other). Though we conceived of these variables as potential mediators, it is possible that they may also function as confounders or as markers of SES.

All statistical analyses were performed using SAS Version 9 · 4 (SAS Institute Inc,.Carry, NC, USA). The approach to model building and the assessment of environmental covariates as potential mediators of the SES-lung function relationship drew on two previous investigations on this subject [5, 9]. In Model 0, we evaluated the relationship of SES to FEV_1_ and FVC percent predicted using linear regression. FEV_1_ percent predicted and FVC percent predicted were not adjusted for age or gender because the regression models used to calculate these values are already age and gender adjusted. However, we did perform a sensitivity analysis including additional adjustment for age and gender to ensure the robustness of our final model. We added the personal smoking variables to Model 0 to form Model 1. We subsequently added covariates to Model 1 to assess the degree to which each exposure attenuated the SES-lung function relationship after adjustment for smoking. To Model 1, we added SHS exposure (Model 2), home biomass use (Model 3), mode of commuting (Model 4), low fruit/vegetable consumption (Model 5), occupational status (Model 6), and all of these covariates (Model 7). The percent explained by a given exposure was calculated by taking the difference in the effect estimate of SES between the base model and a model with an added covariate(s), and then dividing this by the effect estimate of the base model [9, 27]. Model 1 was compared to model 0, whereas models 2–7 were compared to model 1. Additionally, we looked for linear trends in the SES-lung function relationship in each model by treating SES as a continuous variable.

To assess effect modification by age and gender, multiplicative interaction terms along with the main effects were added to the final model. Given evidence that the SES-lung function relationship may differ by age group (18–39, 40–64, ≥ 65 years) [9], we assessed the degree to which age modifies the SES-lung function relationship by testing for an age category (18–39, 40–64, ≥ 65 years)-SES interaction (for this model, SES was treated as continuous). We also performed analyses using our final model stratified by gender and by age category.

## Results

Characteristics of the study population, stratified by SES, are shown in Table 1. The study population had a mean age of 48 and was 53 · 3 % female. A socioeconomic gradient in several baseline characteristics emerged. As SES increased, values for the following variables decreased: mean age; percent female; percent with low fruit/vegetable consumption; percent ever exposed to biomass fuel; pack years of smoking history; the percent walking, biking, or taking a bus without air-conditioning to work; and the percent with retired, household duties, or “worker” occupational categories. Percent commuting via private car and the percent working as a “professional” or “administrator” increased with higher SES. The percent currently smoking, the percent occupied in “services,” and the percent with a history of SHS exposure was similar in the low and intermediate education groups but lower in the high SES group.

**Table 1:** Characteristics of the study population, stratified by SES (*n* = 22, 878), n (%) or mean ± SD

		Education Level		
	Total Population	≤elementary	middle/high	≥college
Subjects, n (%)	22,878 (100)	8375 (36 · 6)	10,351 (45 · 2)	4152 (18 · 2)
Age, years	48 · 4 ± 16 · 4	58 · 5 ± 13 · 3	44 · 9 ± 14 · 6	36 · 8 ± 15 · 1
Female	12,195 (53 · 3)	5048 (60 · 3)	5339 (51 · 6)	1808 (43 · 6)
Smoking				
Current	5249 (22 · 9)	2028 (24 · 2)	2636 (25 · 5)	585 (14 · 1)
Former	878 (3 · 8)	439 (5 · 2)	349 (3 · 4)	90 (2 · 2)
Never	16,751 (73 · 2)	5908 (70 · 5)	7366 (71 · 2)	3477 (83 · 7)
Pack-years^a^	26 · 3 ± 36 · 6	32 · 3 ± 42 · 5	23 · 3 ± 31 · 6	17 · 9 ± 30 · 0
SHS exposed	17,060 (74 · 6)	6492 (77 · 5)	7976 (77 · 1)	2592 (62 · 4)
Biomass ever users	18,214 (79 · 6)	7799 (93 · 1)	8056 (77 · 8)	2359 (56 · 8)
Low fruit/vegetable	13,683 (59 · 8)	5876 (70 · 2)	5886 (56 · 9)	1921 (46 · 3)
Mode of Transport				
None	8 (0 · 0)	6 (0 · 1)	0 (0 · 0)	2 (0 · 1)
Bus w/o AC	2950 (12 · 9)	1157 (13 · 8)	1323 (12 · 8)	470 (11 · 3)
Bus with AC	3665 (16 · 0)	696 (8 · 3)	2011 (19 · 4)	958 (23 · 1)
Scooter	2628 (11 · 5)	691 (8 · 3)	1554 (15 · 0)	383 (9 · 2)
Taxi	128 (0 · 6)	27 (0 · 3)	70 (0 · 7)	31 (0 · 8)
Company Car	330 (1 · 4)	57 (0 · 7)	189 (1 · 8)	84 (2 · 0)
Private Car	1916 (8 · 4)	168 (2 · 0)	929 (9 · 0)	819 (19 · 7)
Train	18 (0 · 1)	0 (0 · 0)	10 (0 · 1)	8 (0 · 2)
Subway	138 (0 · 6)	5 (0 · 1)	59 (0 · 6)	74 (1 · 8)
Walk	3219 (14 · 1)	2246 (26 · 8)	800 (7 · 7)	173 (4 · 2)
Multiple	2709 (11 · 8)	693 (8 · 3)	1247 (12 · 1)	769 (18 · 5)
Bike	5169 (22 · 6)	2629 (31 · 4)	2159 (20 · 9)	381 (9 · 2)
Occupation				
Farmer	110 (0.5)	102 (1.2)	8 (0.1)	0 (0.0)
Worker	1405 (6.1)	689 (8.2)	696 (6.7)	20 (0.5)
Professional	5128 (22.4)	595 (7.1)	2636 (25.5)	1897 (45.7)
Administrator	2969 (13.0)	276 (3.3)	1688 (16.3)	1005 (24.2)
Services	1526 (6.7)	556 (6.6)	909 (8.8)	61 (1.5)
Household	368 (1.6)	206 (2.5)	145 (1.4)	17 (0.4)
Retired	9130 (39.9)	5443 (65.0)	3129 (30.2)	558 (13.4)
Other	2242 (9.8)	508 (6.1)	1140 (11.0)	594 (14.3)
% FEV_1_	97 · 8 ± 16 · 3	96 · 1 ± 18 · 6	98 · 6 ± 14 · 9	99 · 4 ± 14 · 0
% FVC	89 · 3 ± 16 · 1	85 · 4 ± 17 · 5	90 · 8 ± 14 · 8	93 · 5 ± 14 · 4

^a^Pack years excludes never smokers

**Fig. 2:**
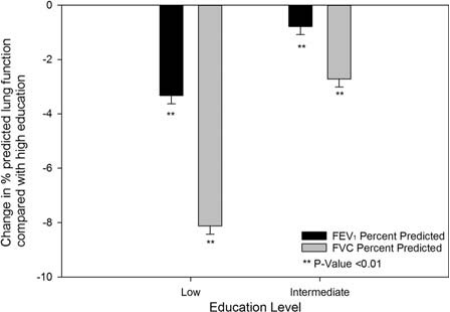
Unadjusted estimates of FEV1 and FVC percent predicted associated with educational level

The unadjusted effect estimates of FEV_1_ and FVC percent predicted (“Model 0”) is shown in Fig. 2. Low education, as compared with high education, was associated with a 3.33 reduction in FEV_1_ percent predicted (95 % CI −3.93, −2.73) and an 8.13 reduction in FVC percent predicted (−8.71, −7.54). Intermediate education, as compared with high education, was associated with a 0.79 reduction in FEV_1_ percent predicted (95 % CI −1.37, −0.21) and a 2.72 reduction in FVC percent predicted (95 % CI −3.29, −2.16).

The results of the multivariate analysis are demonstrated in Table 2. The effect estimates for FEV_1_ and FVC percent predicted for low SES (as compared to high) were negative and statistically significant at *p* <0 · 01 for all models. Additionally, a test for trend (treating SES as a continuous variable) for both FEV_1_ and FVC percent predicted was significant at *P* <0 · 01 for all models. As seen in Table 2, with the exception of SHS, each of the added covariates explained part of the effect of low education on FEV_1_ and/or FVC percent predicted. However, after adjustment for smoking, intermediate education no longer had a significant effect on FEV_1_ percent predicted, and so the “percent explained” for those effect estimates have little meaning; the same is true for the effect of intermediate education on FVC percent predicted in the final model. For the effect of low education on FEV_1_ percent predicted, occupational category had the largest percent explained, followed by smoking, mode of transport, and low fruit/vegetable diet. For the effect of low education on FVC percent predicted, occupational category also had the largest percent explained, followed by mode of transport, biomass exposure, smoking, and finally low fruit/vegetable exposure. For both FEV_1_ and FVC percent predicted, the final model with all covariates included had the greatest percent explained. The final model explained 39 % of the smoking-adjusted reduction in FEV_1_ percent predicted, and 60 % of the smoking-adjusted reduction in FVC percent predicted, that was associated with low education. Of note, in a sensitivity analysis that was additionally adjusted for age and gender, the effect of low education (as compared to high education) on both FEV_1_ and FVC percent predicted was still significant at a p-value < 0.01 in the final model (data not shown).

**Table 2:** Adjusted estimates for percent predicted FEV_1_ and FVC associated with education level

		FEV_1_ % Predicted			FVC % Predicted		
Model	Education	Effect Estimate (95 % CI)	Estimate P-value	% explained	Effect Estimate (95 % CI)	Estimate P-value	% explained
1: Adjusted for smoking	Low	−2.53 (−3.13, −1.94)	<.01	24 %	−7.29 (−7.88, −6.71)	<.01	10 %
	Intermediate	−0.21 (−0.79, 0.37)	0.48	74 %	−2.13 (−2.69, −1.57)	<.01	22 %
	High	Reference	.		Reference	.	
	Trend Test P-Value	<.01			<.01		
2: Model 1 adjusted for SHS	Low	−2.59 (−3.20, −1.99)	<.01	−2 %	−7.37 (−7.96, −6.79)	<.01	-1 %
	Intermediate	−0.27 (−0.85, 0.32)	0.37	−27 %	−2.20 (−2.77, −1.64)	<.01	−4 %
	High	Reference	.		Reference	.	
	Trend Test P-Value	<.01			<.01		
3: Model 1 adjusted for biomass	Low	−2.49 (−3.12, −1.87)	<.01	2 %	−6.31 (−6.92, −5.70)	<.01	14 %
	Intermediate	−0.19 (−0.78, 0.40)	0.54	11 %	−1.56 (−2.13, −0.99)	<.01	27 %
	High	Reference	.		Reference	.	
	Trend Test P-Value	<.01			<.01		
4: Model adjusted for mode of transport	Low	−1.99 (−2.64, −1.33)	<.01	22 %	−5.36 (−5.99, −4.73)	<.01	26 %
	Intermediate	−0.04 (−0.63, 0.55)	0.90	82 %	−1.45 (−2.02, −0.88)	<.01	32 %
	High	Reference	.		Reference	.	
	Trend Test P-Value	<.01			<.01		
5: Model 1 adjusted for low fruit/veg	Low	−2.29 (−2.89, −1.68)	<.01	10 %	−6.86 (−7.45, −6.28)	<.01	6 %
	Intermediate	−0.11 (−0.69, 0.47)	0.70	45 %	−1.96 (−2.53, −1.40)	<.01	8 %
	High	Reference	.		Reference	.	
	Trend Test P-Value	<.01			<.01		
6: Model 1 adjusted for occupation	Low	−1.82 (−2.51, −1.13)	<.01	28 %	−4.05 (−4.72, −3.39)	<.01	44 %
	Intermediate	0.07 (−0.54, 0.67)	0.83	131 %	−0.93 (−1.51, −0.35)	<.01	56 %
	High	Reference	.		Reference	.	
	Trend Test P-Value	<.01			<.01		
7: All Covariates	Low	−1.55 (−2.27, −0.82)	<.01	39 %	−2.93 (−3.62, −2.24)	<.01	60 %
	Intermediate	0.09 (−0.53, 0.70)	0.78	142 %	−0.52 (−1.11, 0.07)	0.08	76 %
	High	Reference	.		Reference	.	
	Trend Test P-Value	<.01			<.01		

Percent explained is the equal to percent reduction in the effect estimate. Model 1 is compared to model 0, and models 2-7 are compared to model 1 (e.g. % explained for model 7 = [model 1 effect estimate – model 7 effect estimate]/model 1 effect estimate). Prediction equations are from an adult Chinese population [25]

Finally, to assess whether the SES-lung function relationship differs across age groups and between genders, the fully adjusted analysis was repeated stratified by age category (Table 3) and by gender (Table 4). The test for trend for the effect of SES on lung function remained significant (*P* < 0.01) for both FEV_1_ and FVC percent predicted for both genders and in all age categories, with the exception of FEV_1_ percent predicted in those aged < 40. The effect of SES on lung function differed both by gender and by age category. In the stratified analysis, a clear gradient in terms of the effect of SES on lung function emerged across the age categories, with a widening in the socioeconomic differential in lung function with increasing age. We found statistically significant effect modification by age category when interaction terms with main effects were added to the final model: the effect of SES on both FEV_1_ and FVC percent predicted was greater for those age > = 65 as compared to both those under 40 years (*P* for interaction < 0.01) and those age 40–64 years old (*P* for interaction < 0.01). We also found effect modification by gender. The deleterious effects of low SES on lung function were greater among men than women in the stratified analysis, and we found that the difference between men and women was statistically significant for both FEV_1_ and FVC percent predicted (*P* for interaction < 0.01).

**Table 3:** Adjusted estimates for percent predicted FEV_1_ and FVC associated with education level stratified by age category

		FEV1 % Predicted^a^		FVC % Predicted^a^	
		Estimate (95 % CI)	P-value	Estimate (95 % CI)	P-value
Age < 40	Low	−0.82 (−2.01, 0.37)	0.18	−1.34 (−2.58, −0.10)	0.03
	Intermediate	−0.58 (–1.25, 0.09)	0.09	−0.77 (−1.47, −0.08)	0.03
	High	Reference	.	Reference	.
	Trend test p-value		0.08		0.01
Age 40 – 64	Low	−1.85 (−3.05, −0.66)	<0.01	−2.23 (−3.39, −1.08)	<0.01
	Intermediate	−0.64 (−1.77, 0.49)	0.27	−0.81 (−1.91, 0.28)	0.14
	High	Reference	.	Reference	.
	Trend test p-value		<0.01		<0.01
Age ≥ 65	Low	−4.76 (−7.33, −2.20)	<0.01	−5.50 (−7.60, −3.40)	<0.01
	Intermediate	−1.65 (−4.39, 1.09)	0.24	−1.42 (−3.67, 0.82)	0.21
	High	Reference	.	Reference	.
	Trend test p-value		<0.01		<0.01

^a^Adjusted for SHS exposure (yes or no), smoking history (current, former, and never), pack years of smoking, biomass exposure (yes or no), low fruit/vegetable diet (<1/day serving of either fruits or vegetables), mode of transport to work, and occupational category

**Table 4:** Adjusted estimates for percent predicted FEV_1_ and FVC associated with education level stratified by gender

		FEV1 % Predicted^a^		FVC % Predicted^a^	
		Estimate (95 % CI)	P-value	Estimate (95 % CI)	P-value
Female	Low	−2.61 (−3.70, −1.52)	<0.01	−3.63 (−4.67, −2.59)	<0.01
(*n* = 12,195)	Intermediate	−0.70 (−1.63, 0.23)	0.14	−1.16 (−2.05, −0.27)	0.01
	High	Reference		Reference	
	Trend test p-value		<0.01		<0.01
Male	Low	−3.66 (−4.63, −2.69)	<0.01	−5.49 (−6.41, −4.57)	<0.01
(*n* = 10,683)	Intermediate	−1.06 (−1.87, −0.24)	0.01	−1.93 (−2.70, −1.15)	<0.01
	High	Reference		Reference	
	Trend test p-value		<0.01		<0.01

^a^Adjusted for SHS exposure (yes or no), smoking history (current, former, and never), pack years of smoking, biomass exposure (yes or no), low fruit/vegetable diet (<1/day serving of either fruits or vegetables), mode of transport to work, and occupational category

## Discussion

In this study, we found evidence for a socio-economic gradient in pulmonary function in an adult Chinese population. We were able to partially explain this association on the basis of several modifiable environmental factors, and we additionally found that the magnitude of the disparities became larger with increasing age. Together, these findings further strengthen long-standing evidence of an SES-lung function relationship, demonstrate that this relationship holds for an adult population in Shanghai across the life course, and suggest that specific life-long environmental factors may possibly play a mediating role.

A relationship between lung function and SES has long been observed. A 2007 review, drawing on 20 studies together involving 125,253 adults and 18,477 children, concluded that SES was an important and underappreciated risk factor for reduced lung function in many countries [1]. While smoking contributes to this relationship, it is “only one piece of the puzzle,” necessitating further investigation into mediating risk factors [1–3]. Outdoor air pollution, indoor air pollution, environmental tobacco exposure, and diet are some additional putative modifiable risk factors. SHS exposure, for instance, emerged as a potential mediator in the SES-lung function relationship among some subgroups in one study [9]. Low SES is also associated with increased exposure to poor outdoor air quality, which could also potentially explain some of the SES-lung function relationship [28]. One study found attenuation in the SES-lung function association after controlling for PM10, something we were unable to do in this study [5]. Dietary factors are another potential mediator given the association between anti-oxidant consumption and lung function [26, 29, 30]. Some have found an attenuation of the SES-lung function relationship when adjusting for vitamin C intake [31], though others have found no mediating effect from low fruit and vegetable consumption [6].

In our study, only some of the SES-lung function relationship was explained by smoking, and, after adjusting for smoking, none was explained by SHS exposure. Occupational category subsequently explained the most of the SES-lung function relationship among the investigated variables. We should note, however, that while this may in part result from differential exposures in the workplace, it may also result from the fact that occupation is itself another index of SES. Some of the “percent explained” of occupation may therefore reflect better adjustment for SES. This may be true, to some extent, of the other covariates, which may function as intermediaries but also as potential confounders or markers of SES. Notably, we also found that a fruit/vegetable deficient diet explained a small part of the relationship. However, perhaps the most novel finding was the identification of mode of transportation to work and exposure to indoor air pollution (biomass exposure) as two important possible mediating factors. These are both plausible mediators from the biological perspective. Biomass use, for instance, is associated with reduced lung function, perhaps in part through the induction of oxidative stress [19]. Commuting exposures, on the other hand, has been associated with varying levels of exposure to ambient pollutants depending on vehicle utilized [32, 33], and has also been associated with adverse short-term respiratory effects [34–36]. Work by our group in this cohort found a relationship between mode of commuting and pulmonary function [21]. However, it is unclear to what extent these differences reflect varying exposure to pollution, routes used, the impact of exercise on minute ventilation and inhaled dose of particles, or other factors.

An unresolved question is the relative importance of prenatal, childhood, and adult exposures in mediating the SES-lung function relationship. For instance, factors affecting intrauterine growth likely have an impact on postnatal lung function [37]. Additionally, when looking at FEV_1_ and FVC in absolute terms (instead of percent predicted), a significant part of the SES-lung function relationship can be explained by the socioeconomic gradient in height, itself the result of early-life exposures, which in turn directly impacts lung function [9, 31]. Social factors in early life may also modify the effect of environmental exposures. For instance, the effect of traffic-related air pollution on lung function may be amplified in households exposed to high levels of stress [38, 39]. However, if the SES-lung function relationship was predominantly the result of intrauterine or childhood exposures, we might expect that the strength of the relationship would either remain stable or be attenuated with increasing age. Conversely, in a recent large cross-sectional study in Scotland, Gray et al. found that the SES-gradient in FEV_1_ actually became larger with increasing age [9]. Our results in this study are consistent with the findings of Gray et al. In stratifying our analysis across the same age categories as Gray et al., we also found that social disparities in lung function widened as age increased, suggesting that ongoing environmental exposures might have a continuing impact on lung function throughout life. An alternative explanation for this finding is that SES was more a determinant of lung function for older cohorts than for younger ones, and therefore reflects the impact of undefined secular environmental trends. However, this would imply analogous secular trends playing out over time in both Scotland and China, which seems less likely. Notably, we also found evidence for effect modification by gender, such that the deleterious effect of low SES on lung function was greater among men than women. We speculate that this could relate to the fact that certain exposures (for instance, in the workplace) may be more associated with low education in men than among women.

We acknowledge some limitations to this study, most notably the cross-sectional design, which prevents us from drawing clear conclusions with respect to causality. However, by using education as a metric for SES, the likelihood of reverse causality is reduced. For instance, the onset of respiratory disease during adulthood could result in reduced SES when measured by current income, but not when measured by previously attained education. Of course, it is possible that children with lung disease might be less likely to pursue higher education. However, if this were the explanation for our findings, we would not expect to see a widening socioeconomic gradient in lung function in older age groups. Additionally, it is worth mentioning that 10,648 individuals declined to participate in the study. It is possible that these individuals differed from those who participated. Notably, a majority of those who declined who were age 18 or over were male, whereas our study population was majority female. This could potentially reflect differing availability to participate given job responsibilities. It is also possible that those who declined may have been of a lower SES (given that written consent was required as part of the study). Even if this were the case, we would expect that the *relationship* between low SES and reduced lung function in these subjects would be similar to the relationship between low SES and reduced lung function in our overall study population. Thus, we would expect that the exclusion of these individuals would only bias our study towards the null. Other potential weaknesses relate to the subjective determination of indoor air pollution, the imprecision of our dietary variable, the lack of information on area level SES, and the lack of data on levels of outdoor or workplace air pollution. It is also important to again emphasize that the covariates we treated as potential mediating factors may actually be functioning as confounders, or – especially in the case of occupation – as markers of SES.

We also recognize several strengths of this study, including its large size and the objective outcome measurement. Additionally, the SES-lung function relationship has been relatively understudied in China, and to our knowledge this is the first study to identify transportation use and indoor air pollution as two potential mediators of this relationship in any country. We believe that this study, in conjunction with others, may have important public health implications. Reducing socioeconomic inequalities in pulmonary health might be approached using two frameworks. First, we can look for mediating factors that produce worse pulmonary health in disadvantaged populations, and then consider targeting those exposures. For instance, public health programs that attempt to address unsafe home cooking fuel use, expand commuting options, modify smoking habits, or increase fruit and vegetable intake among low SES individuals might (and this is admittedly speculative) help to lessen inequalities in pulmonary health. Second, the fact that SES affects pulmonary health even when accounting for known mediating exposures (as was the case in this study) provides a potential argument for going further upstream, and attenuating disparities in socioeconomic conditions themselves. This study provides some tentative support for both frameworks.

## Conclusions

In conclusion, in this study, we found cross-sectional evidence for strong socioeconomic disparities in lung function in Shanghai that widened with increasing age. These disparities were partially attenuated when controlling for several potentially modifiable risk factors, though much of the socioeconomic gradient in lung function remains unexplained. China – like all nations – contends with significant socioeconomic inequalities in health [40]. Further research is needed to further elucidate the respiratory consequences of these disparities.

## Abbreviations

FEV_1_: forced expiratory volume in one second
FVC: forced vital capacity
SES: socioeconomic status
SHS: second hand smoke
